# The Effect of Intracameral Triamcinolone Acetonide on Controlling Common Complications following Phacoemulsification in Dogs

**DOI:** 10.3390/ani14040547

**Published:** 2024-02-07

**Authors:** Zichen Liu, Di Lu, Mo Pang, Jing Li, Yue Liu, Hao Shi, Gang Liu, Yipeng Jin

**Affiliations:** College of Veterinary Medicine, China Agricultural University, No. 2 Yuanmingyuan West Rd, Haidian District, Beijing 100193, China; lzc94@126.com (Z.L.); 15927308865@163.com (D.L.); pangmo1991@126.com (M.P.); dongylijing@163.com (J.L.); liuyue_vip@126.com (Y.L.); haoshi@cau.edu.cn (H.S.); liugang_0402@126.com (G.L.)

**Keywords:** triamcinolone acetonide, glucocorticoid, anterior chambers, phacoemulsification, corneal edema, PGE2, cataract, dogs

## Abstract

**Simple Summary:**

At present, local eye drops, subconjunctival injection, and systemic administration of corticosteroids are the most common methods to control complications in dogs after phacoemulsification. However, these methods are limited by a high frequency of administration, systemic drug reactions in dogs, poor compliance among owners, and high care requirements. In this study, triamcinolone acetonide was injected into the anterior chambers of dogs after phacoemulsification to observe and evaluate its effects on controlling postoperative complications. The intraocular pressure, corneal edema, aqueous flare, aqueous humor protein concentration, and prostaglandin E2 concentration in these dogs were measured and evaluated at different time points before and after surgery. The findings confirmed that the intracameral injection of triamcinolone acetonide achieved a favorable effect on controlling transient ocular hypertension and corneal edema after phacoemulsification in dogs. Moreover, the intracameral injection of 1.5 mg of triamcinolone acetonide decreased the protein and prostaglandin E2 concentrations in the aqueous humor of dogs after surgery. It was demonstrated that triamcinolone acetonide-based treatment can be safely employed to effectively control common complications after phacoemulsification in dogs. Therefore, this treatment method is worthy of clinical application.

**Abstract:**

The intracameral injection of triamcinolone acetonide (TA) has achieved favorable clinical effects in controlling intraocular inflammatory reactions in humans after cataract surgery. However, the effect of this method remains unclear in veterinary practice. In this paper, 18 dogs with bilateral cataracts were randomly divided into three groups, with 6 dogs in each group. Phacoemulsification and intraocular lens implantation were performed on the 36 eyes of these dogs. A total of 0.1 mL of TA solution was injected into the oculus dexter (OD) anterior chambers. All oculus sinister (OS) anterior chambers of these dogs were used as controls. The results demonstrated that the corneal edema severity scores of the OD (1.5 mg TA) were lower than those of the OS from the 1st to 7th day after surgery, with a significant difference on the 3rd day after surgery (*p* = 0.033). The corneal edema severity scores in the OD (1.5 mg TA) were significantly lower than those in the OD (0.5 mg TA) on the 3rd day after surgery (*p* = 0.036). The aqueous humor protein concentration of the OD (1.5 mg TA) had a lower concentration than the OS on the 1st day after surgery (*p* = 0.004). Furthermore, on the 5th and 10th days, the aqueous humor protein concentration of the OD (1.5 mg TA) was lower than that of the OS (*p* = 0.038 and *p* = 0.044, respectively). The aqueous humor PGE2 concentration of the OD (1.5 mg TA) had a lower concentration than the OS on the 1st day after surgery (*p* = 0.026). The aqueous humor PGE2 concentrations in the OD (1.0 mg TA) and OD (1.5 mg TA) were lower compared to that in the OD (0.5 mg TA) on the 1st day after surgery (*p* = 0.041 and *p* = 0.037, respectively). It was demonstrated that TA-based treatment can be safely employed to effectively control common complications after phacoemulsification in dogs.

## 1. Introduction

As a common lens lesion, a cataract can be induced by opacity in the visual axis within the lens. This lesion can seriously affect the vision of animals and even cause blindness [1]. Currently, phacoemulsification is the most common surgical method for the radical treatment of cataracts. After the contents are removed, the intraocular lens (IOL) is implanted into the lens capsule [2]. The vision in 90–95% of dogs can be restored or improved significantly after phacoemulsification [3]. As a result, phacoemulsification has become the gold standard for cataract treatment in both humans and animals.

At present, physical eye injuries have been reduced by cataract surgery to a large extent, owing to the improvement in relevant techniques, instruments, and IOL. Nevertheless, the adhesion and release of inflammatory mediators caused by traumas have still not been eliminated. As an inevitable process of wound healing, this postoperative intraocular inflammatory reaction is conducive to wound repair. However, uncontrolled intraocular inflammation may lead to severe postoperative complications, such as transient ocular hypertension, corneal edema, fibrinous inflammation, synechia, posterior capsule opacification, and secondary glaucoma. Further, traction retinal detachment may be induced in severe cases. These complications can delay visual recovery in affected dogs [4,5].

Recently, clinical control of intraocular inflammation via intracameral triamcinolone acetonide (TA) injection in humans has been reported in many studies. In addition, many studies have confirmed that this method is safe and effective in controlling inflammation after cataract surgery [6]. Mohamed et al. revealed that the concentration of TA in the anterior chamber remained at a high level within 3 weeks after intracameral injection of TA, and TA in the anterior chamber can directly act on the target tissue of the eyes [7,8]. Ozge et al. confirmed that intracameral TA injection during or at the end of surgery can maintain a low level of anterior chamber inflammation and corneal edema on the first day after surgery [9].

As per a 2019 study, there were no significant changes in the intraocular pressure (IOP) and corneal endothelial cell count of the affected eyes after intracameral injection of TA before and after surgery (*p* > 0.05). Compared with the control eyes, the uncorrected visual acuity, best corrected visual acuity, and eye sign scores were significantly improved in the test eyes (*p* < 0.05). Additionally, the concentrations of TNF-α, IL-1β, and IL-6 in the aqueous humor of the test eyes were lower than those of the control eyes, with significant differences (*p* < 0.05). On the 1st and 7th days after surgery, the proportion of affected eyes with mild aqueous flare was higher than that of control eyes, while the proportion of affected eyes with moderate and severe aqueous flare was lower than that of control eyes (*p* < 0.05). Further, the test eyes had a lower incidence of adverse reactions compared with the control eyes [10].

Because TA is a suspension, its special particle structure may lead to an elevated IOP. Karalezli et al. explored the effects of intracameral TA injection on the IOP, and they found that the administration of TA (1 mg) after cataract surgery did not exert significant impacts on the IOP [11]. Li et al. suggested that, after the intracameral injection of TA, the IOP of the affected eyes was high on the 1st day after surgery (up to 24 mmHg), but it could remain stable in the next few days. Additionally, there was no increase in the IOP when the TA dose reached 4 mg [12].

Similarly to humans, dogs can experience many complications induced by phacoemulsification, such as intraocular inflammation, fibrinous inflammation, and corneal edema. These complications affect the recovery of vision after surgery. Effective surgical treatment depends on the control of intraocular inflammation. At present, local eye drops, subconjunctival injection, and systemic administration of corticosteroids are the most common methods used to control complications in dogs after phacoemulsification. However, these methods are limited by a high frequency of administration, systemic drug reactions in dogs, poor compliance among owners, and high care requirements.

In this study, the safety of different doses of TA on the eyes of test dogs and its effects on controlling major complications after phacoemulsification were observed after intracameral TA injections at different doses. Based on this, the effect of intracameral TA injection on the eyes of dogs was evaluated, and the optimal injection dose was also identified. The findings of this study are expected to provide basic data for its subsequent application in veterinary practice.

## 2. Materials and Methods

### 2.1. Animals

In this study, 18 mongrel dogs (aged 1–3 years, weighing 3–10 kg, 8 females and 10 males) were selected as the test animals. They all came from a stray animal rescue station located in Beijing. These test dogs had cataracts in both eyes, and they were all diagnosed with mature cataracts (no systemic disease). No fluorescein staining was found in the corneas of both eyes. There was no significant difference in the Schirmer tear test (STT), IOP, and electroretinogram (ERG) between the oculus dexter (OD) and oculus sinister (OS) of these dogs. The animals displayed no evidence of previous ophthalmic problems, and no medication treatment was in progress before the experiment. These 18 dogs with bilateral cataracts were randomly divided into two groups (TA Group and Control Group), with 18 eyes in each group. The TA Group was further divided into the 0.5 mg TA Group, 1.0 mg TA Group, and 1.5 mg TA Group, with 6 eyes in each group.

### 2.2. Surgical Procedures

**Figure 1:**
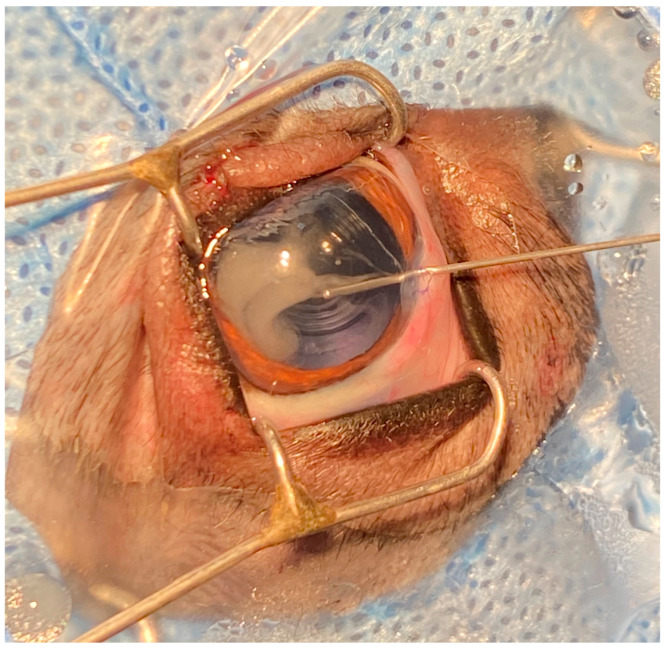
Schematic diagram of TA injection into the anterior chambers during cataract surgery in dogs.

Firstly, phacoemulsification and IOL implantation were performed on the 36 eyes of these dogs. Subsequently, 0.1 mL of perfusion solution (500 mL Sodium Lactate Ringer Injection+ 1 mL Adrenaline Hydrochloride Injection (1 mL:1 mg)+ 1 mL Gentamycin Sulfate Injection (10 mL:0.2 g) +0.16 mL Heparin Sodium Injection (2 mL:12,500 U)) was injected into the OS anterior chambers of these dogs as controls while closing the corneal incision. Meanwhile, TA (1 mL:40 mg) was diluted into a 0.1 mL anterior chamber injection with perfusion solution at the concentrations of 5, 10, and 15 mg/mL and injected into the OD anterior chamber of the 0.5 mg TA Group, 1.0 mg TA Group, and 1.5 mg TA Group, respectively, while closing the corneal incision (Figure 1).

### 2.3. Observation and Measurement of Postoperative Indexes

The IOP, corneal edema, aqueous flare, aqueous humor protein concentration, and aqueous humor prostaglandin E2 (PGE2) concentrations in these dogs were observed and measured after surgery. Specifically, the IOP was measured with the same ophthalmotonometer at the 2nd, 4th, 6th, and 8th hours after surgery and on the 1st, 2nd, 3rd, 4th, 5th, 6th, 7th, 14th, 21st, 28th, and 45th days after surgery. In addition, the distribution of TA particles in the anterior chamber was recorded under the same slit lamp at the 2nd, 4th, 6th, 8th, and 10th hours after surgery. In addition, corneal edema, aqueous flare, and various grading standards were observed on the 1st, 2nd, 3rd, 4th, 5th, 6th, 7th, 14th, 21st, and 28th days and the 2nd month after surgery. Scoring of corneal edema and aqueous flare was graded using the Hackett–McDonald scoring system [13]. Moreover, 0.3 mL of aqueous humor was collected from these dogs during the 4th hour after surgery and on the 1st, 3rd, 5th, and 10th days after surgery. When we collected the aqueous humor, the dogs were fully fixed, and local anesthesia of the eyes (0.5% proparacaine hydrochloride, Alcaine^®^, Alcon, Puurs, Belgium) was applied. For particularly hyperactive dogs, 2.5 mg/kg Zoletil^®^ 50 (Virbac, Nice, France) was injected intravenously, and aqueous humor was extracted after sedation. Then, these aqueous humor samples were stored at −20 °C. These samples were thawed at room temperature and subsequently centrifuged to collect the supernatant for measurement. According to the manufacturer’s instructions, the protein concentration in the aqueous humor samples was detected using a commercially available Pierce BCA Protein Assay Kit (Thermo Scientific, Waltham, MA, USA) [14], and the PGE2 concentration in the aqueous humor samples was detected using a commercially available canine PGE2 Parameter Assay Kit (Jianglai, Shanghai, China) [15].

### 2.4. Data Analysis

This study focused on statistically analyzing the differences in therapeutic effects between the three doses of TA (0.5, 1.0, and 1.5 mg) and their control groups at the same time point, as well as the differences in therapeutic effects among the three doses of TA (0.5, 1.0, and 1.5 mg). The statistical analysis software SPSS Statistics v20 (IBM, New York, NY, USA) was adopted for statistics and data processing. To facilitate the collation and classification of data, this experiment did not adopt a random principle for the selection of test eyes (OD vs. OS). But the data were evaluated by two independent observers blinded to the treatment groups, and the surgical operator did not participate in the scoring. The indexes (IOP, corneal edema, aqueous flare, aqueous humor protein concentration, and aqueous humor PGE2 concentration) of the dogs in each group were expressed as mean ± standard deviation (M ± SD). Student’s *t* test and a repeated measures ANOVA were used for statistical analysis. Additionally, in the R software (version 4.1.2, R Foundation), Tukey’s HSD function was used to check the significance of the above indexes within and between groups at each time point. A *p*-value of <0.05 was considered statistically significant. Groups with significant differences in the table were marked with the same letters. Some data were analyzed and calculated using GraphPad Prism 8 (San Diego, CA, USA) to plot the relevant diagrams.

## 3. Results

### 3.1. Postoperative Indexes

#### 3.1.1. IOP

As described in the previous section, the IOP of the 18 dogs was measured with the same ophthalmotonometer at the 2nd, 4th, 6th, and 8th hours after surgery and on the 1st, 2nd, 3rd, 4th, 5th, 6th, 7th, 14th, 21st, 28th, and 45th days before and after surgery (Table 1).

**Figure 2:**
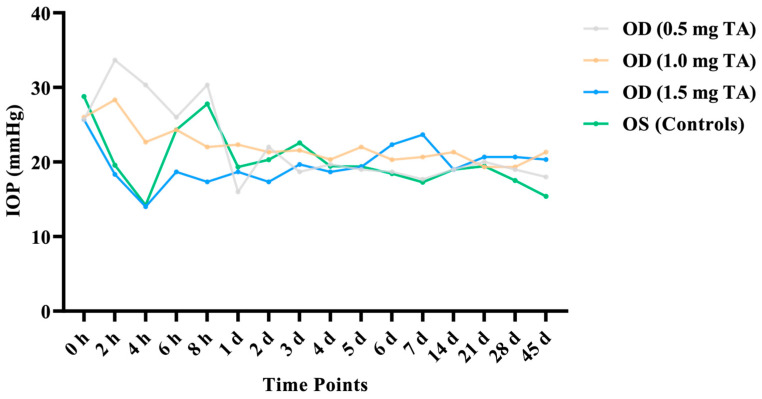
Comparison of the IOP between the TA groups (0.5, 1.0, and 1.5 mg TA) and control groups at the 2nd, 4th, 6th, and 8th hours after surgery and on the 1st, 2nd, 3rd, 4th, 5th, 6th, 7th, 14th, 21st, 28th, and 45th days after surgery.

After phacoemulsification and IOL implantation, the IOP in the OD (0.5 mg TA) increased within 8 h after surgery; the IOP in the OD (1.0 mg TA) increased at the 2nd hour after surgery. Compared with pre-surgery values, the IOP measurements in the OD (1.5 mg TA) and the control eyes were lower at the 4th hour after surgery. The results indicated that there was no significant difference in the binocular IOP at all time points before and after surgery (*p* > 0.05) (Figure 2).

#### 3.1.2. Corneal Edema

According to the corneal edema severity scores, varying degrees occurred in both eyes of the dogs from 1 to 7 days after surgery, and the corneal edema of these dogs disappeared on the 7th day after surgery. The duration of corneal edema after surgery decreased with the increase in TA doses. Specifically, corneal edema in the OD (0.5 mg TA) lasted for 6 days on average and disappeared on the 7th day after surgery; in the OD (1.0 mg TA), corneal edema lasted for 4 days on average and disappeared on the 5th day after surgery; in the OD (1.5 mg TA), corneal edema lasted for 2 days on average and disappeared on the 3rd day after surgery (Table 2).

**Figure 3:**
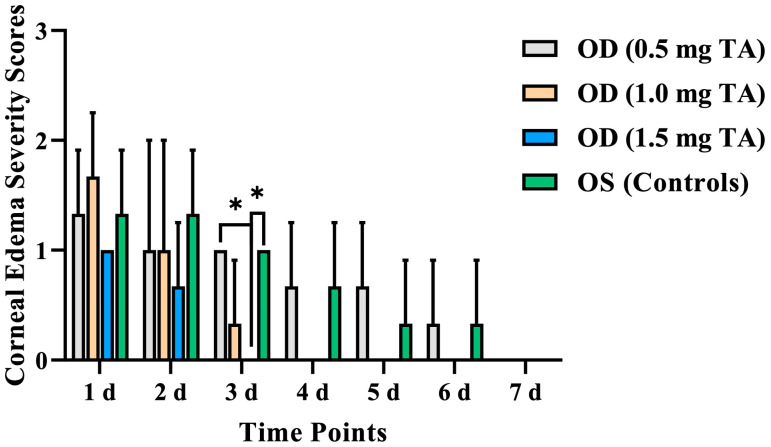
Comparison of the corneal edema severity scores between the TA groups (0.5, 1.0, and 1.5 mg TA) and control groups on the 1st, 2nd, 3rd, 4th, 5th, 6th, and 7th days after surgery. *: *p* < 0.05.

The corneal edema severity scores in the TA groups and control groups reached their peak on the 1st day after surgery. The corneal edema severity scores of the OD (1.5 mg TA) were lower than that of the control eyes from the 1st to 7th day after surgery, with a significant difference on the 3rd day after surgery (*p* = 0.033). The corneal edema severity scores on the 1st day after surgery can be ranked as OD (1.0 mg TA) > OD (0.5 mg TA) > OD (1.5 mg TA). However, the scores at other time points can be ranked as OD (0.5 mg TA) > OD (1.0 mg TA) > OD (1.5 mg TA). Among them, there was a significant difference in the corneal edema severity scores on the 3rd day after surgery between the OD (1.5 mg TA) and OD (0.5 mg TA) (*p* = 0.036) (Figure 3).

#### 3.1.3. Aqueous Flare

There was no aqueous flare in the 36 eyes before surgery, but aqueous flare in varying degrees appeared in all eyes after surgery (Table 3). The aqueous flare in the OD (0.5 mg TA), OD (1.0 mg TA), and OD (1.5 mg TA) resolved within 2.67 ± 2.52, 3.33 ± 1.53, and 2.00 ± 0.00 days, respectively, after surgery. Among them, the duration for the resolution of aqueous flare in the OD (1.5 mg TA) was shorter than those in the OD (0.5 mg TA) and OD (1.0 mg TA). There was no significant difference in the aqueous flare degree and the days for the resolution of aqueous flare between the OD and the control eyes (*p* > 0.05).

### 3.2. Aqueous Humor Analysis

#### 3.2.1. Aqueous Humor Protein Concentration

There was no significant difference in the aqueous humor protein concentrations between the TA groups and control groups before surgery (*p* > 0.05). The aqueous humor protein concentrations in the OD and the control eyes reached the peak at the 4th hour and on the 1st day after surgery. This index in the TA groups and control groups showed a downward trend from the 1st to the 10th day after surgery (Table 4).

**Figure 4:**
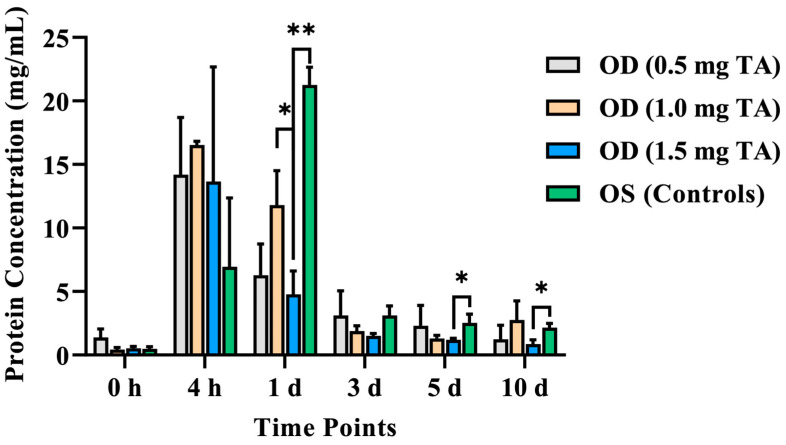
Comparison of the aqueous humor protein concentrations between the TA groups (0.5, 1.0, and 1.5 mg TA) and control groups at the 4th hour after surgery and on the 1st, 3rd, 5th, and 10th days after surgery. *: *p* < 0.05. **: *p* < 0.01.

The aqueous humor protein concentration of the OD (1.5 mg TA) was lower than that of the control eyes from the 1st to 10th day after surgery, with a significant difference on the 1st day after surgery (*p* = 0.004). And this index of the OD (1.5 mg TA) was lower than that of the control eyes from the 1st to 10th day after surgery, with a significant difference on the 5th day (*p* = 0.038) and 10th day (*p* = 0.044) after surgery. There was no significant difference in the aqueous humor protein concentration between the OD groups (*p* > 0.05), except that this index in the OD (1.0 mg TA) was significantly higher than that in the OD (1.5 mg TA) on the 1st day after surgery (*p* = 0.019). Moreover, this index in the OD (1.5 mg TA) was lower than that in the OD (0.5 mg TA) at all time points after surgery, but the difference was not significant (*p* > 0.05) (Figure 4).

#### 3.2.2. Aqueous Humor PGE2 Concentration

As per the canine PGE2 Parameter Assay Kit detection results, there was no significant difference in the aqueous humor PGE2 concentration between the TA groups and control groups before surgery (*p* > 0.05). The aqueous humor PGE2 concentrations in the OD (0.5 mg TA), OD (1.0 mg TA), and the control eyes reached the peak on the 1st day after surgery (206.97 ± 28.72, 121.51 ± 23.88, and 172.48 ± 17.32 pg/mL, respectively). This index in the OD (1.5 mg TA) reached the peak on the 3rd day after surgery (132.55 ± 37.73 pg/mL) and decreased slowly from the 3rd to the 10th day after surgery (Table 5).

**Figure 5:**
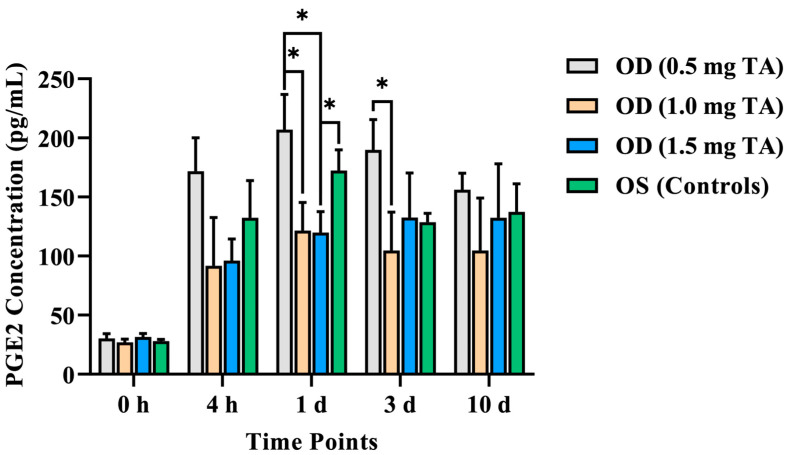
Comparison of the aqueous humor PGE2 concentrations between the TA groups (0.5, 1.0, and 1.5 mg TA) and control groups at the 4th hour after surgery and on the 1st, 3rd, and 10th days after surgery. *: *p* < 0.05.

The aqueous humor PGE2 concentration of the OD (1.5 mg TA) was lower than that of the control eyes, with a significant difference on the 1st day after surgery (*p* = 0.026). The aqueous humor PGE2 concentrations in the OD (1.0 mg TA) and OD (1.5 mg TA) were lower than that in the OD (0.5 mg TA) at the 4th hour after surgery. Compared with the OD (0.5 mg TA), the aqueous humor PGE2 concentrations in the OD (1.0 mg TA) and OD (1.5 mg TA) were lower on the 1st day after surgery, and the differences were also statistically significant (*p* = 0.041 and *p* = 0.037, respectively). This index was also lower in the OD (1.0 mg TA) and OD (1.5 mg TA) compared with the OD (0.5 mg TA) on the 3rd and 10th days after surgery. However, only the OD (1.0 mg TA) was significantly different from the OD (0.5 mg TA) on the 3rd day after surgery (*p* = 0.029). In addition, on the 3rd and 10th days after surgery, this index in the OD (1.5 mg TA) was higher than that in the OD (1.0 mg TA), but there was no significant difference (*p* > 0.05) (Figure 5).

## 4. Discussion

In this study, the IOP of the dogs in the TA groups and control groups decreased after phacoemulsification and IOL implantation, compared with the preoperative value. This may be because the anterior chamber lost the supporting function of the lens and obtained more space after the lens was removed in phacoemulsification. The production of the original aqueous humor cannot maintain the normal IOP. Moreover, there may be chronic iridocyclitis after surgery, and the aqueous humor from the ciliary body may be reduced due to inflammation. Meanwhile, more prostaglandins may be generated due to intraocular inflammation after surgery, which increases the outflow of the aqueous humor and leads to a low IOP.

Local or systemic administration of corticosteroids may induce an increased IOP, which may lead to glaucoma at a later stage [16,17]. In addition, ocular hypertension and corticosteroid-induced glaucoma cannot be observed in the elderly after the intracameral injection of TA (4 mg) at the end of cataract surgery [18]. In another retrospective cohort study, 140 uveitic cataract patients who received phacoemulsification and IOL implantation were reviewed, and the results indicate that intraoperative intravitreal injection of TA (3 mg) caused no significant difference in the mean IOP between the control group at baseline and any postoperative visit [19]. In a veterinary study to determine the ocular safety of a single intravitreal dose of TA (8 mg) (IVTA) in dogs, the authors found that the immediate increase in IOP post-IVTA was short-lived, and the pressure quickly returned to pre-IVTA levels [20]. According to the data from this study, the IOPs in the OD (1.5 mg TA) and the control eyes were lower at the 4th hour after surgery compared with those before surgery. And the IOP in the OD (1.5 mg TA) at all time points after surgery was lower than that before surgery. This indicated that the intracameral injection of TA (1.5 mg) would not lead to corticosteroid-induced transient ocular hypertension. This finding may be related to the balance between the intraocular inflammatory reaction and the anti-inflammatory effect of the drugs. It may be speculated that the intraocular inflammatory reaction can be controlled with the increase in TA doses. In addition, the cellular constituent exudate from the blood–aqueous humor barrier was also reduced, which contributed to a lower occlusion degree of the chamber angle [21].

Corneal edema is one of the most common complications after phacoemulsification. The results of a study based on 507 patients who underwent phacoemulsification revealed that the proportion of patients with corneal edema on the 1st day after surgery reached 87.39% [22]. In this study, the corneal edema severity scores indicated that the duration of corneal edema after surgery in the OD (0.5 mg TA) was longer than that in the OD (1.0 mg TA) and OD (1.5 mg TA). The corneal edema severity scores of the OD (1.5 mg TA) were lower than those of the control eyes and the OD (0.5 mg TA), with a significant difference on the 3rd day after surgery (*p* < 0.05). The corneal edema severity scores 2–6 days after surgery can be ranked as OD (0.5 mg TA) > OD (1.0 mg TA) > OD (1.5 mg TA). This may be related to corneal endothelial cell injury caused by anterior uveitis. With the increase in TA doses, intraocular inflammation was mitigated, and the severity and duration of corneal edema were also reduced. In 2007, Oh et al. performed the intracameral injection of TA in rabbits to identify whether this regimen could cause corneal damage. The results suggested that TA had no significant influence on the corneal central thickness and endothelial cell count, but the number of microvilli decreased [23,24]. Similarly, the results of our study indicated that the intracameral injection of TA would not increase the severity of corneal edema after surgery.

In this study, there was no aqueous flare in any of these dogs’ eyes before surgery. However, all 36 eyes suffered from aqueous flare with varying degrees after surgery. This complication may be caused by mechanical injury and other thermal injuries during surgical procedures that destroyed the blood–aqueous humor barrier. As a result, the cellular and protein constituents leaked into the aqueous humor, which was also the major factor leading to uveitis after phacoemulsification [25,26]. Among them, the duration for the resolution of aqueous flare in the OD (1.5 mg TA) was shorter than that in the OD (0.5 mg TA) and OD (1.0 mg TA). However, there was no significant difference in this index between both groups (*p* > 0.05). This may be because the TA injection of 1.5 mg may be conducive to maintaining the integrity and stability of the blood–aqueous humor barrier. The aqueous flare score in the OD (1.0 mg TA) and OD (1.5 mg TA) was slightly higher than that in the OD (0.5 mg TA) within 1–2 days after surgery. This may be because the TA suspension did not induce a deposition on the ventral side of the anterior chamber within 1–2 days after injection. However, suspended particles indistinguishable from the naked eye may be scattered in the aqueous humor, which increased the aqueous flare score detected based on Tyndall effects. Aqueous humor circulation and venous blood flow in the anterior pigment layer are some of the mechanisms of drug elimination from the aqueous humor [27]. The TA lens persisted in the anterior chamber for several days. It was demonstrated that the half-life of free TA is about 18.7 ± 5.7 days in human eyes without vitrectomy [28]. Currently, there is no relevant report on the retention time of different doses of TA in the anterior chamber after cataract surgery in dogs, which can be further explored in subsequent trials.

There is no fibrinogen and cellulose coagulation in healthy aqueous humor. However, the IOP is temporarily reduced, and the blood–aqueous humor barrier is destroyed during cataract surgery. This causes the accumulation of serum components in the anterior chamber. Among these components, fibrinogen plays a prominent role in triggering fibrin formation, which further leads to fibrin-like reactions in the anterior chamber [5,29]. Wålinder et al. found that 11–17% of the patients who chose extracapsular cataract extraction and IOL implantation had fibrin-like reactions, which usually occurred from the 1st to the 6th day after surgery [30].In this study, the aqueous humor protein concentration of the OD in the TA groups increased and reached its peak at the 4th hour after phacoemulsification and IOL implantation. This was caused by the destruction of the blood–aqueous humor barrier during surgical procedures. Many proteins were gathered in the anterior chamber, thus leading to an increased aqueous humor protein concentration. The aqueous humor protein concentration in all OD groups showed a downward trend from 1 to 10 days after surgery owing to the self-repair of the blood–aqueous humor barrier and the efficacy of TA. Compared with the control eyes, the OD (1.5 mg TA) had the best control effect on anterior chamber protein exudation, significantly improving intraocular inflammation response on the 1st, 5th, and 10th days after surgery (*p* < 0.05).

In addition, the aqueous humor protein concentration of the OD in the TA groups was higher than that of control eyes at the 4th hour after surgery, which may be related to the excipient in commercial TA. In this study, the commercially obtained Transton^®^ (Jida, Kunming, China) is a TA suspension containing excipients. Excipients and particle dispersion in the agent may cause intraocular inflammation [31,32]. Macky et al. proposed that excipients could cause partial toxicity to the eyes of New Zealand white rabbits. In the excipient group, the lens density increased significantly, the amplitude of ERG decreased significantly, and the mitochondria of visible light receptors swelled and ruptured under a transmission electron microscope. On the contrary, all the above examination results in the excipient-free group were normal [33].

As a short-term active lipid mediator, PGE2 is produced via the cyclooxygenase pathway in the irises of dogs and other species. PGE2 has been extensively recognized as a marker of intraocular inflammation in dogs [10,34,35]. According to a previous study [10], increased inflammatory factors in the aqueous humor can not only aggravate the inflammatory reaction but also induce the exudation of cellulose in the anterior chamber, which adheres to the IOL, thus affecting the recovery of postoperative vision. Moreover, excessive accumulation of prostaglandins cannot be eliminated in time after the occurrence of uveitis. The weakened transport mechanism of PGE2 may aggravate the intraocular inflammatory reaction [34]. In this study, the results demonstrated that this index in the TA groups and control groups increased rapidly from 0 to 3 days after surgery, which was higher than the basic value before surgery. In addition, the doses of TA in the OD (1.0 and 1.5 mg) achieved a better effect on inhibiting the aqueous humor PGE2 concentration at the initial stage of inflammation after surgery (within 1 day after surgery) compared with the OD (0.5 mg TA) (*p* < 0.05). Compared with the control eyes, the OD (1.5 mg TA) had the best control effect on the aqueous humor PGE2 concentration, significantly improving intraocular inflammation response on the 1st day after surgery (*p* < 0.05). On the 3rd and 10th days after surgery, the aqueous humor PGE2 concentration in the OD (1.5 mg TA) was higher than that in the OD (1.0 mg TA), but there was no significant difference (*p* > 0.05). This may be because the crystalline TA partially damaged the intraocular tissues (such as the corneal endothelium and iris) during the intracameral injection of TA. Additionally, other components in the drug (such as the excipient) may also exert certain stimulating effects on intraocular tissues.

Currently, it remains unclear whether administering drugs in one eye will affect the contralateral eye of the same individual. In addition, there is a lack of research on the influence of unilateral medication on the contralateral eye after cataract surgery. As per some studies, drugs unilaterally administered into the anterior chamber will not enter the systemic circulation through iris absorption under the integrity of the blood–aqueous humor barrier. Moreover, some viscoelastic agents may remain in the eyes after cataract surgery and partially block the chamber angle, which may induce the accumulation of TA in the eyes, thus achieving a sustained release effect [4]. Therefore, further research is required to identify whether TA will reach and affect the contralateral eye in dogs after phacoemulsification.

There were no reports of using a TA injection into the anterior chamber for canine cataract surgery, so the selection of the TA dose in this experiment was relatively cautious. When designing the experiment, it was considered that excipients and particle dispersion in the agent might cause intraocular inflammation [31,32,33], and there was no relevant report on the retention time of different doses of TA in the anterior chamber after cataract surgery in dogs. At the same time, we referred to the study by Karalezli et al. [11], which found that the administration of TA (1 mg) after cataract surgery did not exert significant impacts on the IOP. Therefore, this experiment only included three doses (0.5, 1.0, and 1.5 mg TA) for research, which had certain limitations and failed to observe the control effect of higher doses of TA (greater than 1.5 mg) on the complications of dog cataract surgery. In future research, further attempts will be made to apply higher TA doses (greater than 1.5 mg) to explore the safety and effectiveness of a TA injection into the anterior chamber. In addition, more dog eye data will be collected as untreated bilateral control groups for statistical analysis, increasing the experimental data.

## 5. Conclusions

These findings confirmed that the intracameral injection of TA could achieve a favorable effect on controlling transient ocular hypertension and corneal edema after phacoemulsification in dogs. In addition, the intracameral injection of 1.5 mg of TA decreased the protein and PGE2 concentrations in the aqueous humor of dogs after surgery. It was demonstrated that TA-based treatment can be safely employed to effectively control common complications after phacoemulsification in dogs.

## Figures and Tables

**Table 1:** IOP (mmHg) values of the 18 test dogs at different time points.

Time Points	TA Group (*n* = 18)			Control Group (*n* = 18)
	OD (0.5 mg TA) (*n* = 6)	OD (1.0 mg TA) (*n* = 6)	OD (1.5 mg TA) (*n* = 6)	OS
0 h	25.67 ± 4.16	26.00 ± 6.08	25.67 ± 2.52	28.77 ± 5.98
2 h	33.67 ± 17.56	28.33 ± 7.02	18.33 ± 0.58	19.58 ± 1.42
4 h	30.33 ± 17.21	22.67 ± 6.35	14.00 ± 3.46	14.20 ± 6.42
6 h	26.00 ± 9.17	24.33 ± 6.35	18.67 ± 1.15	24.31 ± 4.59
8 h	30.33 ± 17.24	22.00 ± 2.65	17.33 ± 1.53	27.77 ± 19.03
1 d	16.00 ± 3.00	22.33 ± 3.51	18.67 ± 0.58	19.32 ± 11.21
2 d	22.00 ± 11.79	21.33 ± 4.04	17.33 ± 2.31	20.31 ± 8.39
3 d	18.67 ± 5.51	21.67 ± 1.53	19.67 ± 1.53	22.57 ± 3.96
4 d	19.67 ± 1.53	20.33 ± 0.58	18.67 ± 1.53	19.43 ± 4.12
5 d	19.00 ± 0.00	22.00 ± 3.00	19.33 ± 0.58	19.39 ± 2.17
6 d	18.67 ± 1.15	20.30 ± 2.89	22.33 ± 2.52	18.43 ± 1.62
7 d	17.67 ± 1.53	20.67 ± 6.66	23.67 ± 7.23	17.29 ± 2.16
14 d	19.00 ± 1.00	21.33 ± 2.52	19.00 ± 2.00	19.00 ± 2.00
21 d	20.00 ± 1.73	19.33 ± 0.58	20.67 ± 1.53	19.44 ± 1.06
28 d	19.00 ± 0.00	19.33 ± 2.52	20.67 ± 1.53	17.53 ± 0.67
45 d	18.00 ± 1.73	21.33 ± 0.58	20.33 ± 1.15	15.40 ± 8.26

TA, triamcinolone acetonide; OD, oculus dexter; OS, oculus sinister.

**Table 2:** Scoring statistics of corneal edema severity of the 18 test dogs at different time points.

Time Points	TA Group (*n* = 18)			Control Group (*n* = 18)
	OD (0.5 mg TA) (*n* = 6)	OD (1.0 mg TA) (*n* = 6)	OD (1.5 mg TA) (*n* = 6)	OS
1 d	1.33 ± 0.58	1.67 ± 0.58	1.00 ± 0.00	1.33 ± 0.58
2 d	1.00 ± 1.00	1.00 ± 1.00	0.67 ± 0.58	1.33 ± 0.58
3 d	1.00 ± 0.00 ^a^	0.33 ± 0.58	0.00 ± 0.00 ^ab^	1.00 ± 0.00 ^b^
4 d	0.67 ± 0.58	0.33 ± 0.58	0.00 ± 0.00	0.67 ± 0.58
5 d	0.67 ± 0.58	0.00 ± 0.00	0.00 ± 0.00	0.33 ± 0.58
6 d	0.33 ± 0.58	0.00 ± 0.00	0.00 ± 0.00	0.33 ± 0.58
7 d	0.00 ± 0.00	0.00 ± 0.00	0.00 ± 0.00	0.00 ± 0.00

TA, triamcinolone acetonide; OD, oculus dexter; OS, oculus sinister; significant differences were observed between groups with the same letter markings.

**Table 3:** Scoring statistics of aqueous flare of the 18 test dogs at different time points.

Time Points	TA Group (*n* = 18)			Control Group (*n* = 18)
	OD (0.5 mg TA) (*n* = 6)	OD (1.0 mg TA) (*n* = 6)	OD (1.5 mg TA) (*n* = 6)	OS
1 d	0.67 ± 0.58	1.33 ± 0.58	1.33 ± 0.58	1.33 ± 0.58
2 d	0.67 ± 0.58	1.33 ± 0.58	1.00 ± 0.00	1.00 ± 1.00
3 d	0.67 ± 0.58	0.67 ± 0.58	0.00 ± 0.00	0.33 ± 0.58
4 d	0.33 ± 0.58	0.33 ± 0.58	0.00 ± 0.00	0.33 ± 0.58
5 d	0.33 ± 0.58	0.33 ± 0.58	0.00 ± 0.00	0.33 ± 0.58
6 d	0.00 ± 0.00	0.00 ± 0.00	0.00 ± 0.00	0.00 ± 0.00
7 d	0.00 ± 0.00	0.00 ± 0.00	0.00 ± 0.00	0.00 ± 0.00

TA, triamcinolone acetonide; OD, oculus dexter; OS, oculus sinister.

**Table 4:** Protein concentrations (mg/mL) in the aqueous humor of the 18 test dogs.

Time Points	TA Group (*n* = 18)			Control Group (*n* = 18)
	OD (0.5 mg TA) (*n* = 6)	OD (1.0 mg TA) (*n* = 6)	OD (1.5 mg TA) (*n* = 6)	OS
0 h	1.38 ± 0.67	0.43 ± 0.17	0.54 ± 0.13	0.49 ± 0.16
4 h	14.20 ± 4.50	16.53 ± 0.29	13.66 ± 9.02	6.94 ± 5.43
1 d	6.29 ± 2.45	11.80 ± 2.71 ^a^	4.79 ± 1.82 ^ab^	21.27 ± 1.39 ^b^
3 d	3.12 ± 1.94	1.88 ± 0.43	1.50 ± 0.20	3.11 ± 0.76
5 d	2.31 ± 1.59	1.31 ± 0.24	1.20 ± 0.13 ^c^	2.53 ± 0.69 ^c^
10 d	1.23 ± 1.15	2.76 ± 1.51	0.87 ± 0.33 ^d^	2.16 ± 0.32 ^d^

TA, triamcinolone acetonide; OD, oculus dexter; OS, oculus sinister; significant differences were observed between groups with the same letter markings.

**Table 5:** PGE2 concentrations (pg/mL) in the aqueous humor of the 18 test dogs.

Time Points	TA Group (*n* = 18)			Control Group (*n* = 18)
	OD (0.5 mg TA) (*n* = 6)	OD (1.0 mg TA) (*n* = 6)	OD (1.5 mg TA) (*n* = 6)	OS
0 h	30.35 ± 3.82	26.95 ± 2.7	31.61 ± 2.93	27.94 ± 1.62
4 h	171.70 ± 28.4	91.67 ± 40.99	96.24 ± 18.19	132.41 ± 31.52
1 d	206.97 ± 28.72 ^ab^	121.51 ± 23.88 ^a^	119.80 ± 17.85 ^bc^	172.48 ± 17.32 ^c^
3 d	189.83 ± 25.77 ^d^	104.64 ± 32.65 ^d^	132.55 ± 37.73	128.66 ± 7.56
10 d	156.13 ± 13.92	104.67 ± 44.36	132.40 ± 45.67	137.49 ± 23.58

TA, triamcinolone acetonide; OD, oculus dexter; OS, oculus sinister; significant differences were observed between groups with the same letter markings.

## Data Availability

The data presented in this study are available in the article.
